# Enhanced photoreduction CO_2_ efficiency by criss-crossed TiO_2_ nanoflakes combined with CdS under visible light

**DOI:** 10.1098/rsos.181789

**Published:** 2019-03-13

**Authors:** Fei Wang, Juangang Wang, Yunhuan Cheng

**Affiliations:** Anhui Key Laboratory of Energetic Materials, College of Chemistry and Material Science, Huaibei Normal University, Huaibei 235000, Anhui, People's Republic of China

**Keywords:** rapid propagation of charge carriers, criss-crossed TiO_2_ nanoflakes, photoreduction CO_2_

## Abstract

In this paper, a novel photocatalyst CNCC with excellent visible light photocatalytic performance was successfully prepared to optimize the CO_2_ photoreduction performance. The results showed that the methanol formation rate of CNCC was 24.7 µmol g^−1^ h^−1^, which was 1.42 times higher than that of NCC. The enhanced photoactivity is attributed to the rapid propagation of charge carriers induced by light from the constructed composite structure.

## 1. Introduction

Photocatalysis is increasingly being seen as a potential alternative to solar fuel production [1–4]. In addition to the formation of hydrogen from water, another focus is photocatalytic reduction in CO_2_. Because of the technical difficulties associated with hydrogen storage, products [5–8] from CO_2_, such as methane and methanol, can easily be used as current energy sources. It is well known that one of the most significant challenges in photoelectrochemical processes is highly efficient separation and transmission of photoinduced electron–hole pairs. To suppress a recombination and improve transmission of electron–hole pairs, researchers have designed many schemes. A novel photocatalyst Ag_3_PO_4_@MWCNTs@PANI and Z-scheme heterojunction photocatalyst Ag_3_PO_4_@MWCNTs@Cr:SrTiO_3_ were successfully prepared by a facile *in situ* precipitation method [9,10]. The MWCNTs penetrating in the bulk phase of Ag_3_PO_4_ could serve as conductors of photogenerated electrons and rapidly migrated electrons to the surface of the photocatalysts. Han *et al*. [11] synthesized uniform spherical CdS/TiO_2_ core–shell nanoparticles with different TiO_2_ shell thicknesses, generating an energy gradient at the interface to spatially separate the electrons and holes. Zhan *et al*. [12] have prepared TiO_2_ nanorod films on FTO substrates, which exhibit a longer electron lifetime and more effective separation of photogenerated electron–hole pairs. A highly efficient Pt–TiO_2_ nanostructured film, with fast electron-transfer rate and efficient electron–hole separation by the Pt nanoparticles, is reported [13]. In this paper, we propose a material model of criss-crossed TiO_2_ nanoflakes combined with CdS (CNCC) and compared its photocatalytic activity with that of the usual TiO_2_ nanoparticles with CdS (NCC). The geometry structure of criss-crossed TiO_2_ nanoflakes is conductive to charge separation, therefore inhibiting recombination can significantly improve its CO_2_ photoreduction performance.

## 2. Experimental methods

All chemicals used in the experiment (Tianjin Chemical Reagent Company) are analytical reagent grade.

The FTO glass (5 × 5 cm^2^) was cleaned, and then coated with TiO_2_ nanowire in ethanol solution. FTO glass was placed in a tetrafluoroethylene reactor (200 ml), 110 ml ethanol, 5 ml deionized water, 1 mmol Ti(OC_2_H_5_)_4_, 1 mmol trihydroxytriethylamine, 1 mmol urea glycoside and 1 mmol hexadecanol were added, and then placed in an oven at 180°C for 7 days. The substrate was washed and baked at 450°C for 50 min to optimize the photoreduction performance of CO_2_.

The CdS–TiO_2_ heterostructure photocatalysts were prepared using a simple precipitation method. The FTO film of prepared interconnected TiO_2_ nanowires was dispersed in a certain volume of 0.1 M Cd (NO3)_2_ aqueous solution, using the same capacity of 0.1 M Na_2_S aqueous solution as the precipitator introduced by dripping slowly. Then the film was rinsed several times with deionized water. The anatase TiO_2_ nanoparticles (15 nm primary particle size) were purchased from Aladdin. The synthetic procedures of TiO_2_ nanoparticles combined with CdS were almost the same as the procedures of criss-crossed TiO_2_ nanoflakes combined with CdS.

## 3. Results and discussion

### 3.1. Morphological characteristics and phase structures

**Figure 1.**
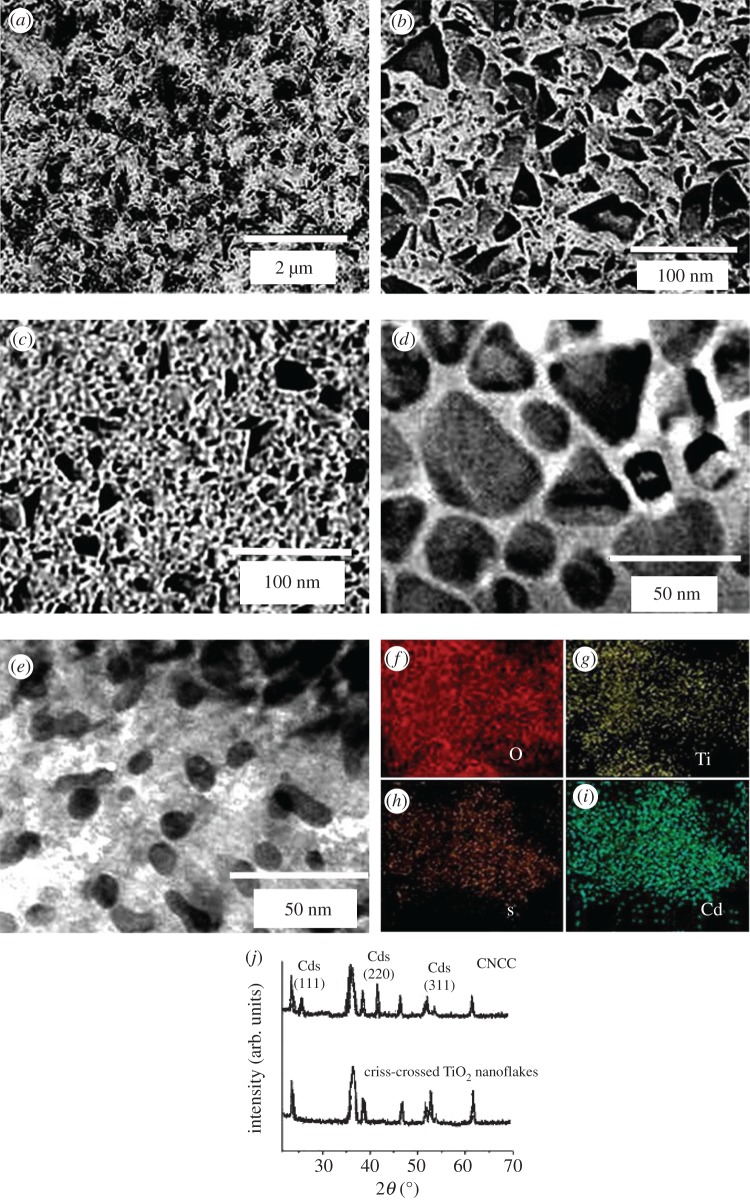
Morphology of the CNCC. (*a*) SEM image of the top view of the criss-crossed TiO_2_ nanoflakes. (*b*) A magnified SEM image of the criss-crossed TiO_2_ nanoflakes (*c*) A magnified FESEM image of the CNCC. TEM images of TiO_2_ (*d*) and TiO_2_–CdS (*e*) composite. (*f–i*) Composed elemental mapping image. (*j*) XRD pattern of the CNCC and the criss-crossed TiO_2_ nanoflakes.

The morphology of TiO_2_ on FTO glass was studied by SEM. Figure 1*a* indicates the micrometre-sized TiO_2_ film. A magnified SEM image in figure 1*b* clearly demonstrates the highly organized structure of the film packaging by criss-crossed TiO_2_ nanoflakes, which provide more surface area and active sites for the next catalytic reaction [14,15]. Figure 1*c* displays magnified SEM of the criss-crossed TiO_2_ nanoflakes covered with CdS-nanosized crystallites. The CdS particle size is about 8 nm. Transmission electron microscopy (TEM) images for TiO_2_ and TiO_2_–CdS composite are presented in figure 1*d*,*e* to explore the nanostructure. As shown in figure 1*d*, criss-crossed nanoflakes were distinctly observed. From figure 1*e*, it can be seen that the porous surface is composed of nanoparticles with a diameter of less than 10 nm. EDX mapping images (figure 1*f–i*) indicate that the sample contains Ti, O, Cd and S; this finding further confirms the coexistence of titanium dioxide and cadmium sulfide. Ti, O, Cd and S are well dispersed in the samples; thus, CdS nanoparticles are uniformly dispersed on the surface of TiO_2_. Their phase structures were studied by XRD. Figure 1*j* shows the XRD diagram of criss-crossed TiO_2_ nanoflakes, indicating formation of the anatase phase. The diffraction peak of TiO_2_ can be seen by observing the XRD diagram of CdS@TiO_2_ composite. The peaks located at 26.5°, 44.4° and 52° could be indexed to the (111), (220) and (311) crystal planes of cubic CdS phase, respectively.

### 3.2. Transportation time and recombination time constants

**Figure 2.**
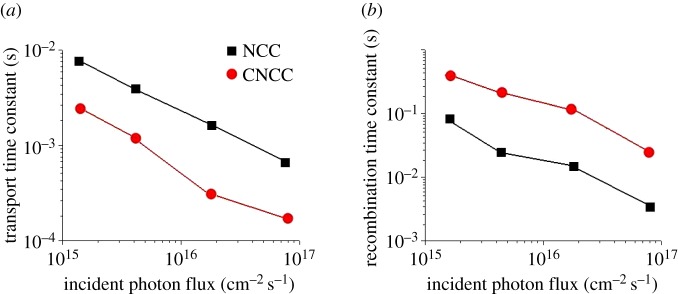
Comparison of transmission (*a*) and recombination (*b*) time constants for CNCC and NCC.

The transmission and recombination of photoinduced electrons are the main determinants of the efficiency of CO_2_ photoreduction; thus, the study of these effects in CNCC is of great significance for the further development of the CO_2_ photoreduction process. Intensity-modulated photocurrent spectroscopy was used to measure the transmission characteristics and intensity-modulated photovoltage spectroscopy was used to measure recombination characteristics [16]. Figure 2*a* compares the transmission time constants of TiO_2_ nanoflakes and nano-titanium dioxide particles combined with CdS as light intensity functions. The transmission time constant *τ*_c_ of CNCC is 2.24 × 10^−4^ s at the light intensity (9.12 × 10^16^ cm^−2^ s^−1^) and 3.31 × 10^−3^ s at the light intensity (1.15 × 10^15^ cm^−2^ s^−1^). Meanwhile, the transmission time constant *τ*_c_ of NCC is 8.34 × 10^−4^ s at 9.19 × 10^16^ cm^2^ s^−1^ and 8.04 × 10^−3^ s at 1.18 × 10^15^ cm^−2^ s^−1^. The electron transmission between TiO_2_ nanoparticles and CdS is slower than that of CNCC film; this may be due to the electron's residence time in the trap of the particle network (for example, the number of connections between particles) and the region of contact between particles that limits it [17]. That is to say, the criss-crossed TiO_2_ nanoflakes are a fine electrical conductive body along the orientation of the strip axes relative to TiO_2_ nanoparticles. Figure 2*b* shows the recombination time constant of NNCC is two or three orders of magnitude larger than that of NCC in the studied range of light intensity. Slower carrier recombination indicates the criss-crossed TiO_2_ nanoflakes have less surface recombination sites than TiO_2_ nanoparticles.

### 3.3. Photocatalytic reduction activity

**Figure 3.**
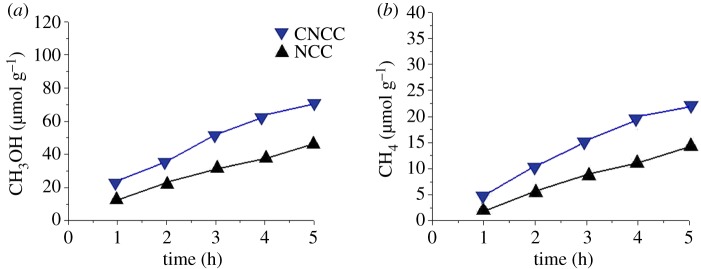
Yield of (*a*) CH_3_OH and (*b*) CH_4_ upon photoreduction in CO_2_ as a function of time under visible light by using CNCC and NCC.

A self-made 100 ml quartz reactor was used for photocatalytic CO_2_ reduction. A 400 W xenon lamp with a 420 nm cut-off filter was used as the optical source. Gas products from photocatalytic reduction of CO_2_ were collected with a 1 µm syringe and analysed rapidly by the gas chromatograph (SP-7890) with a flame ionization detector. Detailed experimental procedures of photocatalytic CO_2_ reduction can be found in supplementary materials. Figure 3 presents the methanol and methane formation rates of NCC and CNCC in 5 h of irradiation. For CNCC and NCC, it is noteworthy that the yield of methanol (figure 3*a*) is much higher than that of methane (figure 3*b*). It can be seen that CNCC achieves a methanol formation rate of 24.7 µmol g^−1^ h^−1^, 1.42 times higher than that of NCC. The results show that the methane production rate of CNCC was 4.59 µmol g^−1^ h^−1^, 1.68 times higher than that of NCC. The geometry structure of criss-crossed TiO_2_ nanoflakes can significantly improve its CO_2_ photoreduction performance. When visible light irradiates the photocatalyst, CdS acts as a sensitizer at this time, converting the TiO_2_ response from ultraviolet to visible light. Photoinduced electrons from CdS to the conductive bands of TiO_2_ can reduce carbon dioxide to negative electrodes, resulting in the formation of methane or methanol. The electron transmission between criss-crossed TiO_2_ nanoflakes and CdS is faster than that of NCC film, thereby inhibiting recombination with holes.

## 4. Conclusion

The criss-crossed TiO_2_ nanoflakes combined with CdS were successfully synthesized by a simple solvothermal method using hexadecanol as hydrophobic modifier. The CNCC composites exhibited good CO_2_ reduction photocatalytic activity under visible light. The results showed that the methane production rate of CNCC was 4.59 µmol g^−1^ h^−1^, which is 1.68 times higher than that of NCC. CNCC achieves a methanol formation rate of 24.7 µmol g^−1^ h^−1^, which is 1.42 times higher than that of NCC. The increase in CH_4_ and CH_3_OH yields can be attributed to the fast electron–hole transmission of TiO_2_ nanoflakes. This provides a new photocatalytic method for the efficient conversion of CO_2_ by solar energy.

## Supplementary Material

Photocatalytic reduction activity

Reviewer comments

## Data accessibility

Complete data for photocatalytic CO_2_ reduction are available from the Dryad Digital Repository: https://doi.org/10.5061/dryad.r2f59f0 [18].

## References

[1] Herzog H 2011 Scaling up carbon dioxide capture and storage: from megatons to gigatons. Energy Econ. 33, 597–604. (10.1016/j.eneco.2010.11.004)

[2] Guo Q, Zhang Q, Wang H, Liu Z, Zhao Z 2016 Core-shell structured ZnO@Cu-Zn–Al layered double hydroxides with enhanced photocatalytic efficiency for CO_2_ reduction. Catal. Commun. 77, 118–122. (10.1016/j.catcom.2016.01.019)

[3] Ye L, Wu D, Chu K, Wang B, Xie H, Yip H, Wong P 2016 Phosphorylation of g-C_3_N_4_ for enhanced photocatalytic CO_2_ reduction. Chem. Eng. J. 304, 376–383. (10.1016/j.cej.2016.06.059)

[4] Fujiwara H, Hosokawa H, Murakoshi K, Wada Y, Yanagida S, Okada T, Kobayashi H 1997 Effect of surface structures on photocatalytic CO_2_reduction using quantized CdS nanocrystallites. J. Phys. Chem. B 101, 8270–8278. (10.1021/jp971621q)

[5] Akple M, Low J, Qin Z, Wageh S, Al-Ghamdi A, Yu J, Liu S 2015 Nitrogen-doped TiO_2_ microsheets with enhanced visible light photocatalytic activity for CO_2_ reduction. Chin. J. Catal. 36, 2127–2134. (10.1016/S1872-2067(15)60989-5)

[6] Paulino P, Salim V, Resende N 2016 Zn-Cu promoted TiO_2_ photocatalyst for CO_2_ reduction with H*_2_*O under UV light. Appl. Catal. B 185, 362–370. (10.1016/j.apcatb.2015.12.037)

[7] Tahir M, Amin N 2013 Advances in visible light responsive titanium oxide-based photocatalysts for CO_2_ conversion to hydrocarbon fuels. Energy Convers. Manage. 76 194–214. (10.1016/j.enconman.2013.07.046)

[8] Ehsan M, Ashiq M, He T 2015 Hollow and mesoporous ZnTe microspheres: synthesis and visible-light photocatalytic reduction of carbon dioxide into methane. RSC Adv. 5, 6186–6194. (10.1039/c4ra13593h)

[9] Lin Y, Wu S, Yang C, Chen M, Li X 2019 Preparation of size-controlled silver phosphate catalysts and their enhanced photocatalysis performance via synergetic effect with MWCNTs and PANI. Appl. Catal. B. 245, 71–86. (10.1016/j.apcatb.2018.12.048)

[10] Lin Y, Wu S, Li X, Wu X, Yang C, Zeng G, Peng Y, Zhou Q, Lu L 2018 Microstructure and performance of Z-scheme photocatalyst of silver phosphate modified by MWCNTs and Cr-doped SrTiO3 for malachite green degradation. Appl. Catal. B 227 557–570. (10.1016/j.apcatb.2018.01.054)

[11] Han S, Pu Y-C, Zheng L, Zhang J, Fang X 2015 Shell-thickness dependent electron transfer and relaxation in type-II core–shell CdS/TiO_2_ structures with optimized photoelectrochemical performance. J. Mater. Chem. A 3, 22 627–22 635. (10.1039/c5ta07100c)

[12] Zhan F, Liu W, Li H, Yang Y 2018 Ce-doped CdS quantum dot sensitized TiO_2_ nanorod films with enhanced visible-light photoelectrochemical properties. Appl. Surf. Sci. 455, 476 (10.1016/j.apsusc.2018.05.226)

[13] Wang W-N, An W-J, Ramalingam B, Mukherjee S, Niedzwiedzki D, Gangopadhyay S, Biswas P 2012 Size and structure matter: enhanced CO_2_ photoreduction efficiency by size-resolved ultrafine Pt nanoparticles on TiO_2_ single crystals. J. Am. Chem. Soc. 134, 11 276–11 281. (10.1021/ja304075b) 22694165

[14] Wu S, He H, Li X, Yang C, Zeng G, Wu B, He S, Lu L 2018 Insights into atrazine degradation by persulfate activation using composite of nanoscale zero-valent iron and graphene: performances and mechanisms. Chem. Eng. J. 341, 126–136. (10.1016/j.cej.2018.01.136)

[15] Wu S, Li H, Li X, He H, Yang C 2018 Performances and mechanisms of efficient degradation of atrazine using peroxymonosulfate and ferrate as oxidants. Chem. Eng. J. 353, 533–541. (10.1016/j.cej.2018.06.133)

[16] Vande L, Frank A 2001 Nonthermalized electron transport in dye-sensitized nanocrystalline TiO_2_ films: transient photocurrent and random-walk modeling studies. J. Phys. Chem. B 105, 11 194–11 205. (10.1021/jp0118468)

[17] Fabregat-Santiago F, Garcia-Belmonte G, Mora-Sero I, Bisquer J 2011 Characterization of nanostructured hybrid and organic solar cells by impedance spectroscopy. Phys. Chem. Chem. Phys. 13, 9083–9118. (10.1039/C0CP02249G) 21468446

[18] Wang F, Wang J, Cheng Y 2019 Data from: Enhanced photoreduction CO_2_ efficiency by criss-crossed TiO_2_ nanoflakes combined with CdS under visible light *Dryad Digital Repository*. (10.5061/dryad.r2f59f0) PMC6458405 31032034

