# Supplementary material for: Enhanced photoreduction CO2 efficiency by criss-crossed TiO2 nanoflakes combined with CdS under visible light
Source: R Soc Open Sci. 2019 Mar 13;6(3):181789. doi: 10.1098/rsos.181789 (PMC6458405; doi:10.1098/rsos.181789)
Supplement: Photocatalytic reduction activity [file rsos181789supp1.doc]

Photocatalytic CO_2_ Reduction

In the photocatalytic reduction experiment of CO_2_, the FTO film was suspended in 100mL water on vigorous magnetic stirring, and high purity CO_2_ gas was continuously bubbled through the solution at the rate of 0.5mL/min. A 400W Xe lamp, out putting the light density of about 100m W/cm^2^, was used as the light source. The photocatalytic CO_2_ reduction experiments were conducted in a closed Quartz reactor at 0 °C. Prior to irradiation, CO_2_ gas was bubbled to the solution for 30 min to eject the dissolved oxygen in the Quartz reactor. The Quartz reactor temperature was kept at about 0 °C by recirculating cooling water system to increase the solubility of CO_2_ and also to dissipate the heat generated during the irradiation of suspension. The liquid products were qualitatively analyzed by SP-7890 on chromatography system at the flow rate of 1mL/min at column temperature of 30°C through detecting and matching the chromatographic peaks with those for the authentic formate standard.
